# Why Natural or Electron Irradiated Sheep Wool Show Anomalous Sorption of Higher Concentrations of Copper(II)

**DOI:** 10.3390/molecules23123180

**Published:** 2018-12-02

**Authors:** Mária Porubská, Angela Kleinová, Peter Hybler, Jana Braniša

**Affiliations:** 1 Constantine the Philosopher University in Nitra, Faculty of Natural Science, Department of Chemistry, Tr. A. Hlinku 1, 94974 Nitra, Slovakia; jbranisa@ukf.sk; 2 Polymer Institute, Slovak Academy of Sciences, Dúbravská cesta 9, 84541 Bratislava 45, Slovakia; Angela.Kleinova@savba.sk; 3 PROGRESA FINAL SK, s.r.o., Ferienčíkova 18, 81108 Bratislava-Staré Mesto, Slovakia; peter.hybler@progresafinal.sk

**Keywords:** sheep wool, electron irradiation, copper sorption, copper complexes, clusters

## Abstract

Sorption of higher concentrations of Cu(II) solution onto natural sheep wool or wool irradiated by an electron beam was studied. Sorption isotherms were of unexpected character, showing extremes. The samples with lower absorbed doses adsorbed less than non-irradiated wool, while higher doses led to increased sorption varying with both concentration and dose. FTIR spectra taken from the fibre surface and bulk were different. It was concluded that there was formation of Cu(II)-complexes of carboxylic and cysteic acids with ligands coming from various keratin macromolecules. Clusters of chains crosslinked through the ligands on the surface limit diffusion of Cu(II) into the bulk of fibre, thus decreasing the sorption. After exhausting the available ligands on the surface the remaining Cu(II) cations diffuse into the keratin bulk. Here, depending on accessibility of suitable ligands, Cu(II) creates simple or complex salts giving rise to the sorption extremes. Suggestion of a mechanism for this phenomenon is presented.

## 1. Introduction

Many biopolymers find use in separative technology as adsorbents of either plant [1,2,3,4,5] or animal origin [6,7,8,9,10,11,12]. Both groups of biopolymeric adsorbents have been obtained from waste material after being treated using some suitable modifying techniques of chemical or physical character.

As regards adsorbents based on animal products, the most frequently mentioned are sheep wool or colloidal keratin, as well as chitosan. Wool keratin in particular contains several types of functional groups capable of interacting with many chemical species. Radetić et al. [6] tested the sorption of Pb(II) onto recycled wool based on nonwoven material modified with plasma or chitosan. The sorption was qualified as a diffusion-controlled process matching a linearized Freundlich isotherm. In a subsequent work [7] similar adsorbents were used to examine the sorption of some divalent metal cations such as Pb(II), Cu(II), Zn(II) and Co(II) under different concentration, temperature and pH conditions. The best sorption results were found with hydrogen peroxide-treated wool in the order Pb(II) > Cu(II) > Zn(II) > Co(II) when given in mg/g. However, this order changed to Cu(II) > Zn(II) > Pb(II) > Co(II) when the uptake was recalculated as mmol/g. Manassra et al. [8] utilized wool-packed columns to remove either Cr(VI) or Cr(III) from aqueous solutions using different eluent pH values. A low pH of 1 was effective for Cr(VI) uptake and higher pH was better for Cr(III). Hanzlíková et al. [9] mentioned sorption of Cr(III) onto wool irradiated by an electron beam, finding a positive effect of absorbed dose. Sorption of Cu(II) onto wool was investigated by Sheffield et al. [10] applying various experimental conditions. The copper uptake increased with pH up to pH 6 with further pH increases leading to the precipitation of solid copper salts. Naik et al. [11] conducted experiments with wool powders. Although wool fibre powdering led to a negligible change in the surface area, the sorption capacities for Co(II), Cu(II) and Cd(II) were significantly higher. Examining only Co(II) sorption onto wool powder, Wen et al. [12] found the correspondence with the Langmuir isotherm indicating a monolayer Co(II) covering. In general, besides concentration of the tested metal cations in the solutions, the wool sorbent performance showed a dependence on contact time, pH and temperature of the bath, as well as the presence of competitive cations. 

Recently Hanzliková et al. [13] published a study on the sorption of Cr(III), Cd(II) and Pb(II) onto sheep wool irradiated by an accelerated electron beam with absorbed doses from 0 to 350 kGy. The individual applied concentrations were up to 1.4 mmol·dm^−3^. Similar to what was mentioned above, no sorption decrease was observed but, on the contrary, the irradiated wool showed an increasing sorption tendency. In addition, a unified Cr(III) concentration of 24 mmol·dm^−3^ was tested for each absorbed dose using VIS spectrometry. For such a Cr(III) concentration the wool sorption capacity attained a stable value from a dose around 50 kGy or above. 

The comparison of the sorption effectivity of various bioadsorbents is problematic since many authors present only relative data on the removal of cations instead of absolute figures. In addition, there are data mentioning that the sorption percentage decreases with the increasing cation concentration in the bath. That is a logical result considering curvature and levelling of the isotherms. Furthermore, a common feature of the published works is the application of initial cation concentrations in solution below 10 mmol·dm^−3^. With the exception of a slight decline in Zn- and Co-isotherms for the concentration around 4 mmol·dm^−3^ indicated in [7], maybe within an experimental deviation, no authors have mentioned any minimum/maximum values in the isotherm runs. According to our knowledge no extreme was observed within the most often used isotherm models [14,15] applied to the low concentrations. On the other hand, no author has dealt with a metal cation sorption applying higher concentrations, probably due to decreasing sorption efficiency. However, polymer adsorbents can be put into the recovery processes with various concentrations of the components to be recycled. That is why we were interested in the interactions of keratin biopolymer with higher amounts of a metal cation.

Since wool modified with electron beam, as a novel adsorbent, has been investigated relatively little, in this work we have focused on the sorption of Cu(II) cation onto such treated wool applying higher Cu(II) concentrations and trying to understand the relevant sorption mechanism. The obtained findings could be exploited in optimization of wool radiation modification as well as recycling conditions.

## 2. Results and Discussion

Variations of primary and secondary structures of wool by the electron beam effect depending on absorbed dose was published by Porubská et al. [16]. The exposure led to disruption of disulphide bonds, generation of S-sulphonate, cysteine mono- and dioxide with step by step transformation into cysteic acid. Changes for alkyl groups and secondary structure conformation were observed, too. In addition, a positive effect on sorption properties of the irradiated wool was found [9,13].

### 2.1. Sorption of Cu(II)

**Figure 1:**
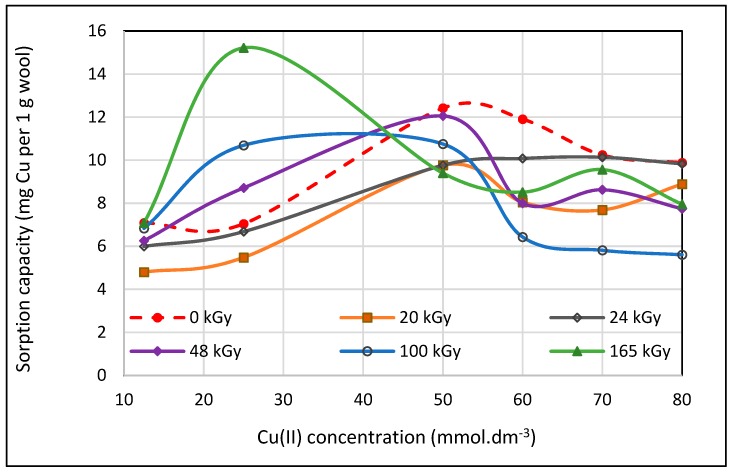
Variations of Cu(II) sorption onto irradiated sheep wool depending on concentration and absorbed dose.

We examined the effect of the wool irradiation on the wool sorption capacity for Cu(II) using multiple higher Cu(II) concentrations than described by other authors. For concentrations on the order of tenths of millimol (mM) per dm^−3^ we observed unexpected courses of the sorption isotherms including extremes, regardless of the absorbed dose (Figure 1). The position of the extremes (the maximum or minimum) is not uniformly related to the concentration or absorbed dose. This variability has to be a consequence of some electron beam effect on the structural properties of the wool. To explain the observed courses of the isotherms we applied firstly infrared spectroscopic analysis.

### 2.2. FTIR Analysis of Wool After Contact with Cu(II)

**Figure 2:**
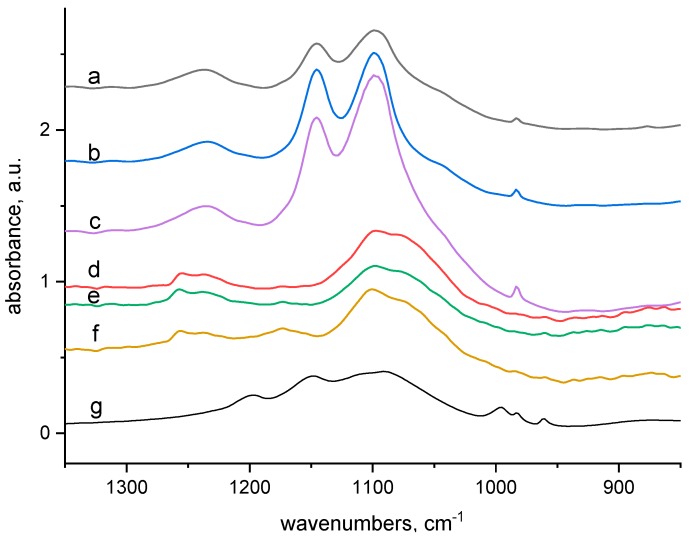
Comparison of the transmission and ATR spectra of wool with absorbed doses 0 kGy, 24 kGy and 165 kGy respectively after contact with 100 mmol·dm^−3^ CuSO_4_ solution (region 900–1300 cm^−1^); from top to bottom: (a), (b) and (c) transmission spectra of 0 kGy, 24 kGy and 165 kGy dosed samples; (d), (e) and (f)—ATR spectra of 0 kGy, 24 kGy and 165 kGy dosed samples, g)—spectrum of CuSO_4_.

**Figure 3:**
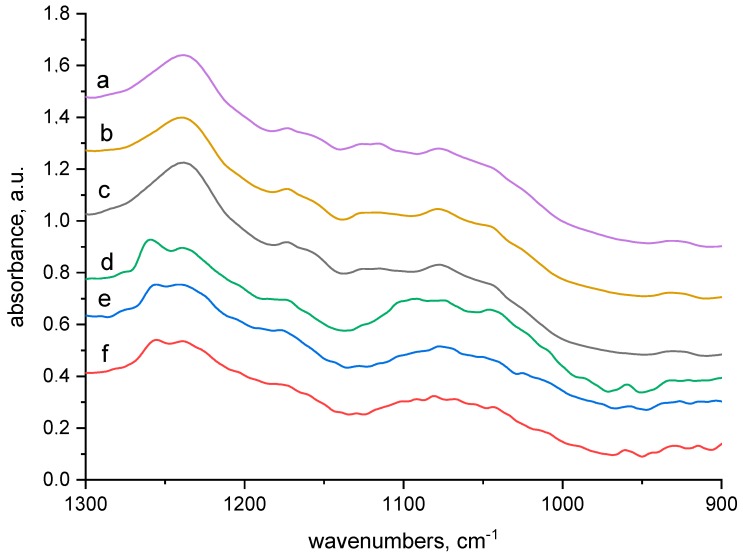
Spectra of reference materials (without CuSO_4_ treatment), from up to down: (a) transmission spectrum of wool with 0 kGy dose; (b) transmission spectrum of wool with 24 kGy dose; (c) transmission spectrum of wool with 165 kGy dose; (d) ATR spectrum of wool with 0 kGy dose; (e) ATR spectrum of wool with 24 kGy dose; (f) ATR spectrum of wool with 165 kGy dose.

**Figure 4:**
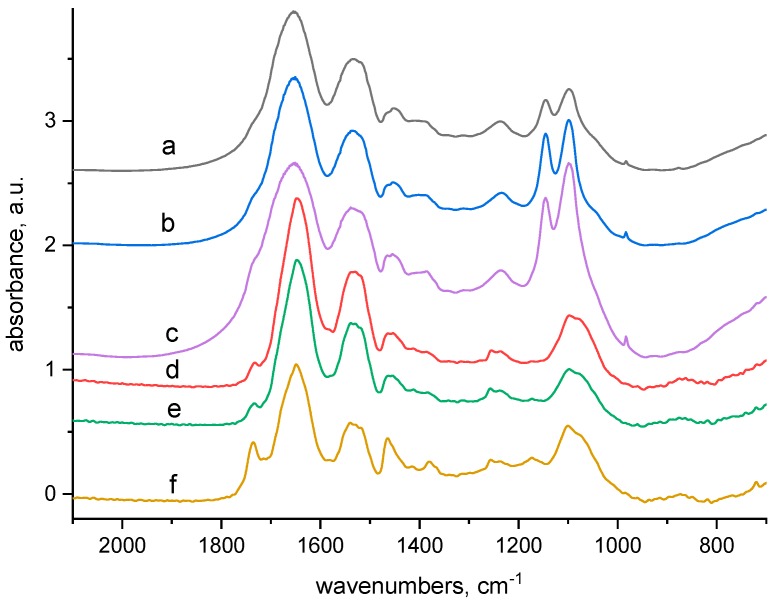
Comparison of the transmission and ATR spectra of wool with absorbed doses 0 kGy, 24 kGy and 165 kGy respectively after contact with 100 mmol·dm^−3^ CuSO_4_ solution (region 700–2000 cm^−1^); from top to bottom: (**a**–**c**)—transmission spectra of 0 kGy, 24 kGy and 165 kGy dosed samples; (**d**–**f**)—ATR spectra of 0 kGy, 24 kGy and 165 kGy dosed samples.

**Figure 5:**
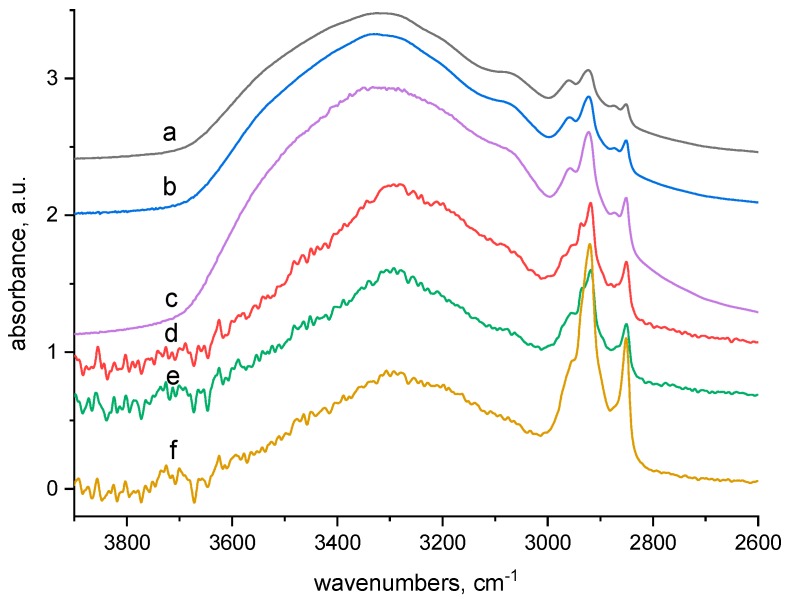
Comparison of the transmission and ATR spectra of wool with absorbed doses 0 kGy, 24 kGy and 165 kGy respectively after contact with 100 mmol·dm^−3^ CuSO_4_ solution (region 3800–2600 cm^−1^); from top to bottom: (**a**–**c**)—transmission spectra of 0 kGy, 24 kGy and 165 kGy dosed samples; (**d**–**f**)—ATR spectra of 0 kGy, 24 kGy and 165 kGy dosed samples.

We used FTIR spectra taken from both the surface (ATR) and bulk (transmission) of the dried wool after 24 h of wool contact with 100 mM CuSO_4_ solution. Analysing them we took into consideration the chemical structure, involving amide, amine, imine, carboxyl, hydroxyl and disulphide groups, as well as considerable tendency of Cu(II) to create complexes. There are appropriate conditions for formation of complexes Cu(II)-wool. To examine if and how the electron beam modification influences the formation of the complexes in the wool, we analysed the wool samples with absorbed doses of 0–24–165 kGy before and after contact with 100 mM CuSO_4_ solution without any bath treatment. The corresponding transmission and ATR spectra in selected regions are displayed in Figure 2, Figure 3, Figure 4 and Figure 5.

Searching the spectra in the 900–1300 cm^−1^ region (Figure 2 and Figure 3) it can be seen that the vinyl (ethylene) group –CH=CH_2_ (978–984 cm^−1^) (Figure 2a–c) occurs inside the samples but is not observable on the surface (Figure 2d–f). At 1098 and 1147 cm^−1^ (Figure 2a–c) a strong sulphate doublet is seen, while on the surface spectra (Figure 2d–f) a partial overlapping of the main SO_4_^2−^ band with either the HSO_4_^−^ band (1078 cm^−1^) or with cysteine monoxide [16,17,18] is seen. However, the adjacent sulphate band at 1147 cm^−1^ is not observable. This indicates, that on the fibre surface such prevalence of HSO_4_^−^ along with a small amount of SO_4_^2−^ exists so that the band 1147 cm^−1^ already lapses. A small band at 1175 cm^−1^ (Figure 2e,f) rising with the dose shows the presence of sulphonic (cysteic) acid [18] but, it is not evident in the fibre bulk. However, it is observable in all spectra corresponding to the samples without Cu(II) contact (Figure 3a–f), so we are inclined to believe that the ATR band at 1175 cm^−1^ (Figure 2d–f) belongs to the overlapping of cysteic acid with HSO_4_^−^ ion [17], too. A band observed in the wool bulk with no sorbate around 1240 cm^−1^ (Figure 3a–c) corresponds to the amide III band (mixed NH vibrations). In the relative ATR spectrum this band separates into two, with peaks at 1255 (irradiated wool) and 1236 cm^−1^ (Figure 3d–f). In the unexposed wool without the sorbate the higher peak is observed at 1260 cm^−1^ (Figure 3d). The higher 1260 or 1255 cm^−1^ peak, but missing inside the fibre, indicates an α-helical conformation and the lower one at 1236 cm^−1^ should correspond to a mixture of β-sheet and disordered structure [19]. The morphology of this doublet varies mildly with dose and Cu(II) contact; absorbance ratio A_1260_/A_1236_ of the bands in doublet for unexposed wool is clearly different from A_1255_/A_1236_ for 24 and 165 kGy dosed wools (Figure 3e,f). However, the differences are less evident after contact with CuSO_4_ (Figure 2d–f). Analogous variations in this doublet morphology were presented also under thermal loading of keratin nanofibres up to 180 °C [20]. As presented by some authors [19,21] the character of this band is sensitive to secondary structure changes. In a pioneering study Porubská et al. [16] showed a severe effect of electron beams on the secondary structure of wool. Low doses up 60 kGy led to a considerably variable distribution of particular conformations (α-helix, β-sheet and amorphous phase) while a clear predominance of β-sheet and amorphous phase over the α-helical one was observed for higher doses. Therefore, we assigned the variation of the doublet at 1255 and 1236 cm^−1^ (Figure 2 and Figure 3) to modification of the secondary structure. As mentioned by Jackson and Mantsch [22] the character of this band depends on keratin side branches and H-bonds. Just such dramatic variations in the secondary structure statistically increase the probability of breaking of existing bonds and formation of new ones, including the binding of amine groups with carboxylate or S-oxidized groups.

The absorption around 1385 cm^−1^ (Figure 4) corresponds to alkyl groups (C-CH_3_) and to coupled C-O valence vibrations with O-H deformation vibrations but also to carboxylate anion. In the ATR spectra (Figure 4d–f) a weak peak at 1412 cm^−1^ indicates carboxylate –COO^−^, too. Since in the transmission spectra (Figure 4a–c) it is not differentiated in such a way, the carboxylate concentration is probably lower in the fibre bulk. Bands in the region from 1454 to 1470 cm^−1^ belonging to alkyl groups occur in all spectra (Figure 4) however, their ratio is different. In the ATR spectrum for the 165 kGy sample (Figure 4f), there are only 1465 cm^−1^ band profiles, apparently demonstrating a definite reorientation of the alkyl groups, unlike for 0 and 24 kGy dosed samples. A small band at 1730 cm^−1^ indicating presence of carboxylic acid moieties is evident in the ATR spectra of all samples (Figure 4d–f). In the corresponding bulk transmission spectra this band is not differentiated. Details of the secondary amide II band (1530 cm^−1^) in the ATR spectra for samples dosed at 24 and 165 kGy (Figure 4e,f) are a little more structured in comparison with the corresponding transmission spectra (Figure 4b,c) indicating that surface and bulk of the fibre do not interact identically. This supports the opinion mentioned above that secondary structure of the amide II group on the fibre surface has been modified.

Alkyls and also the OH bound in carboxylic acids through intramolecular H-bonds with > CO absorb in the region from 2850 to 2970 cm^−1^. Figure 5 displays a considerable structuring of the surface spectrum in this region which is even more evident than at 1445 to 1470 cm^−1^. This points to a larger variety in the wool-Cu(II) interactions and supports the idea of changes in the spatial orientation of the alkyl components of ligands, which has to be accompanied by a corresponding H-bond modification, too. Comparing transmission (Figure 5a–c) with ATR spectra (Figure 5d–f) it can be seen that elimination of CH_3_ valence vibrations around 2874 and 2960 cm^−1^ occurs practically on the fibre surface with the 165 kGy dose, while the band of CH_2_ valence vibrations at 2920 cm^−1^ seems to be unchanged (Figure 5f). In the transmission spectra of all samples (Figure 5a–c) the valence vibrations of free secondary amides (3070 cm^−1^) [17,18] are noticeable but, it is not so in the ATR spectra (Figure 5d–f). This suggests that amides on the fibre surface are partially coupled with Cu(II). Comparison of the ratio for the bands around 3300 cm^−1^ (free amines) to the adjacent group of the alkyl bands (2850–2970 cm^−1^) in the ATR spectra points to a decrease in the valence vibrations of free amine bonds (3300 cm^−1^) on the fibre surface. The decrease can be explained by involvement also of the amine portion on the surface in the interaction with Cu(II) and it ceasing to be free. The band disproportion increases with absorbed dose. For the 165 kGy dosed sample (Figure 5f) the inequality is even more evident than for 24 kGy sample, indicating an intensive amine-Cu(II) interaction on the 165 kGy-irradiated fibre surface. Analogously, a milder variation in the absorbance ratio of the amine band to alkyl groups is observable inside the fibre too (Figure 5a–c). This indicates that complexes formed with participation of amine ligands are generated across the fibre volume being a function of dose.

In work [13], concerning formation of model Cu-complex in solution of CuSO_4_ and arginine HOOC-CH(NH_2_)-(CH_2_)_3_-NH-C(NH_2_)=NH, it was demonstrated that arginine is relatively abundantly present in the side branches of keratin. Mixing of the components immediately led to a change of colour from pale blue (CuSO_4_) to rich aquamarine (CuSO_4_ + arginine). In the corresponding VIS spectrum this product showed a blue shift in comparison with CuSO_4_ absorption. Cu(II) as a borderline Lewis acid accepts electrons from Lewis bases such as –NH_2_, =NH, –OH, and =CO available in keratin. Since wool keratin contains quite a high number of acid and basic functional groups, this provides the best conditions to complex Cu(II) in keratin. In addition, treatment of wool with an electron beam increases the volume of acid groups generating, inter alia, cysteic acid through splitting of S-S bridges and oxidation processes [16,20] and, in this way, supports formation of Cu(II) salts. The question is why FTIR spectra from the fibre bulk and surface indicate different interactions of Cu(II) with wool regardless of whether the wool was irradiated or not. 

The explanation can be provided by the work of Zimmerman et al. [23] which identified trends of localization of electric charge linked to scale on merino wool surfaces using Atomic Force Microscopy (AFM). It was found that on selected surface localities different charge values occurred which correlates well with differing chemical compositions of wool. All scales had a net surface charge negative in sign, however, certain scales had more of negative charged groups on their surface than others. Such an observation shows a good correlation with the distribution of residues from acid amino acids and these can come only from aspartic or glutamic acid. Groups of carboxyl acids were distributed in small clusters uniformly on the fibre surface, while amine groups were localized nearby the scale edge (lysine or arginine). In total, however, the density of the groups resulted in a net average negative charge on the whole surface. If this is so, it is reasonable that Cu(II) cations will immediately adhere to the negative charged surface of scales and get into the sphere of influence of carboxyl or cysteic acids with subsequent Cu-salt creation. Neighbouring amine-groups will have a tendency for the Cu(II)-salt to chelate at once. Regarding the cluster distribution of carboxylic acids on the fibre surface, a higher density of the Cu-salt will be in the cluster locality and consumption of ligands to form chelates will increase there, too. Ligands (amine, imine, hydroxyl) for one Cu(II)-salt exhausted in the locality in question can be provided from several rather than one keratin molecule. In this way through ligands, crosslinking between several chains can be created, forming clusters of nodal points. These clusters of crosslinks present a spatial barrier for the diffusion of further Cu(II) ions into the fibre bulk. Consequence there is a decrease of Cu(II) sorption by the wool manifested by a decreasing sorption capacity despite a higher Cu(II) concentration in the bath. The next increase CuSO_4_ concentration in the bath can, in terms of Fick laws [24], enhance diffusion of further Cu(II) ions into fibre bulk increasing again the wool sorption capacity. If further chelates are created in the fibre bulk from ligands occurring in the force field of Cu(II) salt (e.g., Cu-carboxylate or Cu-cysteinate), the chelate barrier effect can repeat, so despite higher Cu(II) concentration in the bath, a decrease in sorption is observed again (Figure 1). In addition, it is obvious that individual variations in primary and secondary structure of the wool due to various absorbed doses have to change the Cu(II) sorption efficiency.

**Figure 6:**
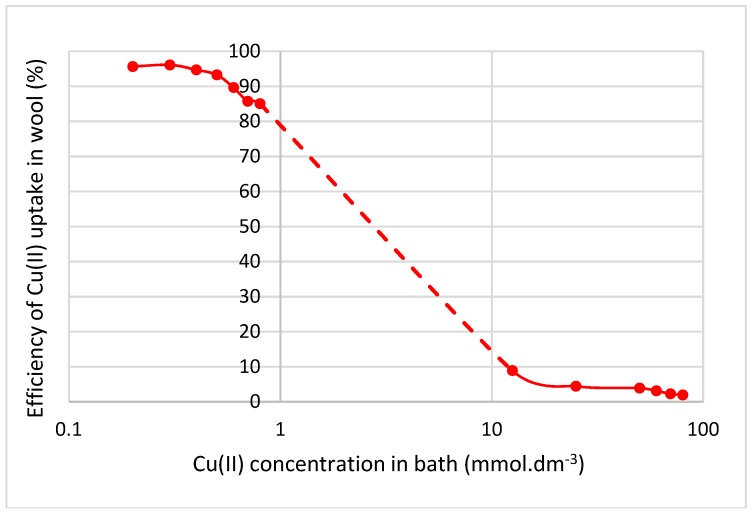
Efficiency variation of Cu(II) uptake by wool depending on Cu(II) concentration in bath for non-irradiated wool.

A different situation is applicable for baths with low CuSO_4_ concentrations. Then the small number of the formed crosslinkings, if any, is still negligible and the sorption efficiency is high. Therefore, the value of information on sorption efficiency (or removal of a cation from solution) is low and misleading without mention of the initial concentration of the bath. Figure 6 shows how this parameter varies with Cu(II) concentration for non-radiated wool. The dependence correlates well with the sorption mechanism proposed above.

### 2.3. EPR Spectrometry

The EPR spectra of wool treated in solutions containing cupric ions exhibited multicomponent anisotropic signals (spectra not shown). However, exact quantification of individual sub-spectra contributions to the resulting EPR spectrum was impossible to achieve. In addition, the spectra showed angular-dependent signal and therefore it was not possible to extract the spin Hamiltonian parameters from the spectra either by computer simulation or manual subtraction. The g-factors were observed to be in the range 2.0–2.25. The EPR spectra contained multicomponent features due to the cupric ion (d^9^ system, S = 1/2) with partially resolved hyperfine splitting patterns (nuclear spin of copper(II) I = 3/2). Irradiated wool contained various deprotonated functional groups of amino acids which may serve as potential donor atoms to form bonds with cupric ions. Based on the EPR spectra it can be proposed that cupric ions form complexes with ionized carboxylate groups, amino groups or thiol groups of cysteine. Copper(II) complexes often exhibit a distorted tetrahedral or octahedral structure [25]. Pentacoordinate copper(II) complexes are more rarely encountered. In this case, the Cu(II) coordination polyhedron is a square pyramid or less frequently a trigonal bipyramid. Based on the observed EPR spectra (g-factors) we may conclude that the coordination environment around copper(II) ion is distorted octahedral or tetrahedral. Even at high gain we were unable to record half-field transitions [26,27], which points to a relatively high (> 10Å) distance between neighboring copper-copper ions. From the shape of the EPR spectra we cannot exclude formation of dimeric, tetrameric or polymeric structures, most probably formed via carboxylate bridges [28,29]. 

The character of EPR spectra gives indirect evidence corresponding to the FTIR findings on abundant presence of complex species. Then based on FTIR and EPR analyses the following processes can take place in the cupric bath (W = wool):

W-R_1_COOH + H_2_O ⇆ W-R_1_COO^−^ + H_3_O^+^ (1)

W-R_2_SO_3_H + H_2_O ⇆ W-R_2_SO_3_^−^ + H_3_O^+^ (2)

CuSO_4_ ⇆ Cu^2+^ + SO_4_^2−^ (3)

SO_4_^2-^ + H_3_O^+^ ⇆ HSO_4_^−^ + H_2_O (4)

Cu^2+^ + 2 W-R_1_COOH + 2H_2_O ⇆ (W-R_1_COO)_2_Cu + 2H_3_O^+^ (5)

Cu^2+^ + 2 W-R_2_SO_3_H + 2H_2_O ⇆ (W-R_2_SO_3_)_2_Cu + 2H_3_O^+^ (6)

W-R_1_COO^−^ + W-R_2_SO_3_^−^ + Cu^2+^ ⇆ (W-R_1_COO)(W-R_2_SO_3_)Cu (binary salt) (7)

Assumed complexes:

(W-R_1_COO)_2_Cu + *x* W-R_3_NH_2_ ⇆ [Cu(W-R_3_NH_2_)_x_ ](COOR_1_-W)_2_ (8)

(W-R_2_SO_3_)_2_Cu + y W-R_4_NH_2_ ⇆ [Cu(W-R_4_NH_2_)_y_ ](SO_3_R_2_-W)_2_ (9)

or in general: [Cu(W-R_1_COO)_a_. (W-R_2_SO_3_)_b_. (W-R_3_NH_2_)_c_] (10)

In Equations (8)–(10) the R-NH_2_ can be replaced by R-OH, R_1_R_2_>C=NH or R-CO-NH- and the corresponding products would be of various structures. The reason for lower sorption capacity of wool with low doses 20 and 24 kGy in comparison with 0 kGy sample (Figure 1) still remains to be interpreted.

### 2.4. Assumed Reasons for the Reduced Sorption

As mentioned above, in the low dose range dramatic changes in both chemical and secondary structures take place in wool and primary S-oxidized species such as S-sulphonate R-S-SO_3_^–^, cystine monooxide R-S-SO-, cystine dioxide R-S-SO_2_- are generated [16]. In these products the S-O bonds are strongly polarized toward oxygen so that they are the strong hydrogen bond acceptors [30]. Whereas the surface layers of the fibre are richer in sulphur content than the inner ones, the abundance of S-oxidized species will be near the surface and these can modify the overall surface electric charge. Within the first phase of sorption this is important because it decides the degree of Cu(II) adhesion to the fibre. According to our hypothesis just change of the electric charge occurs in low dosed samples so that the Cu(II) adhesion to the wool declines or even there is some repulsion. Then the result is lower sorption of Cu(II) in these samples than in the unexposed wool. 

**Figure 7:**
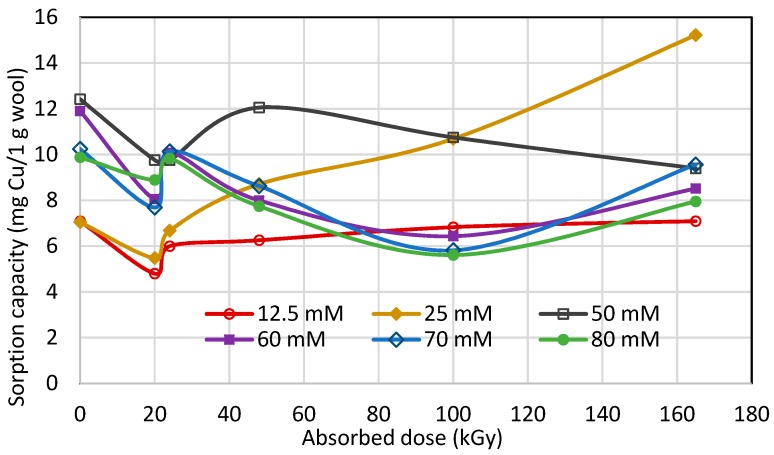
Variation of Cu(II) sorption onto irradiated sheep wool depending on absorbed dose for different Cu(II) bath concentration.

**Figure 8:**
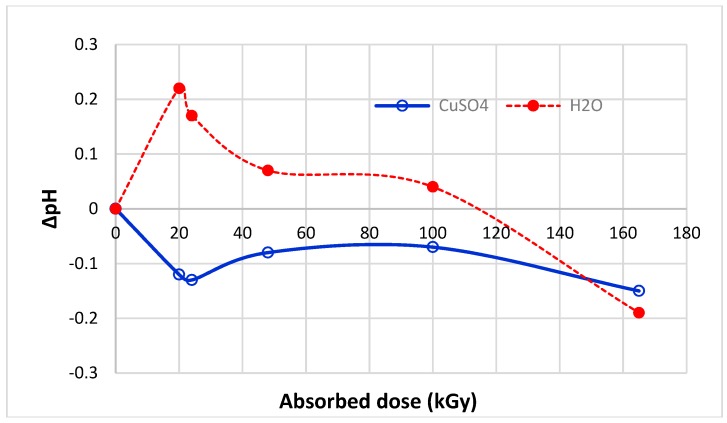
Development of pH-differences in baths containing wool with different absorbed dose immersed in deionized water or 100 mmol.dm^−3^ CuSO_4_ against wool with 0 kGy.

The sorption isotherms (Figure 1) transformed into dependence of the sorption on absorbed dose for the particular Cu(II) concentrations offers interesting evidence (Figure 7); firstly, all curves show a sudden turn at 20 kGy dose; secondly, in consideration of the 0 kGy absorbed dose, the curves can be divided into two groups: (a) the curves for lower concentrations (12.5 and 25 mM) and the ones for higher concentrations (50 to 80 mM). Regarding time-dependent structural variations described by Hanzlíková et al. [31] and providing that the structure has already been stabilized approximately, in this range of 20 kGy dose the amount of disulphide S-S bonds should be changed only a little. At the same time, the amount of S-sulphonate $-S-{\mathrm{SO}}_{3}^{-}-$, cystine monoxide –SO-S- and carboxylate –COO^−^ run through a minimum and the presence of cystine dioxide -SO_2_-S- is negligible in practice. However, the content of cysteic acid increased to an enormous degree up-to an approximate order of 2–3. Despite the stoichiometric conditions to create cysteic-Cu(II) salt in 20 kGy dosed wool, the observed sorption is lower at all applied Cu(II) concentrations than for 0 kGy sample. We deduced that some modification of the wool surface charge can come into consideration and it could be manifest by the pH value. Therefore, the pH-value both of deionized water and Cu(II) bath (100 mM concentration) after contact with the samples was measured. Differences between the pH values of the baths for all dosed samples and the 0 kGy dosed sample are displayed in Figure 8. 

As it can be seen, a notable deviation for 20 and 24 kGy is common for both the sorption capacity (Figure 7) and the pH variations (Figure 8). Pure deionized water showed pH = 6.22 and 100 mM CuSO_4_ solution pH = 4.22. In the bath with 0 kGy dosed wool the water pH decreased to 5.88 but, in the bath with CuSO_4_ a higher pH = 4.31 was measured. We explain the water pH decrease by dissociation of keratin R-COOH in water releasing H_3_O^+^ (Equation (1)). In presence of CuSO_4_, hydrolysis of SO_4_^2−^ occurs consuming H_3_O^+^ so that pH of the initial CuSO_4_ solution increases (Equation (4)). 

Opposite development of pH than the bath with 0 kGy wool is shown for the irradiated samples (Figure 8). It is evident that for 20 and 24 kGy dosed wool the corresponding ΔpH is the highest within doses (0–100) kGy. In the case of CuSO_4_ the pH value dropped by 0.12 or 0.13 against the 0 kGy sample and the bath acidified which can be caused only by higher production of H_3_O^+^. In this way competition between Cu^2+^ and H_3_O^+^ ions towards carboxylate anion is enhanced resulting in lower Cu(II) sorption onto 20 or 24 kGy wool than 0 kGy, resp. Furthermore, Cu(II) comes into coordination with the present amine (amide, imine, hydroxyl) groups binding them as ligands and so formation of zwitterions R-(COO)(NH_3_^+^) is restricted. Thus more separable H^+^ ions result from carboxylic groups able to acidify the bath. Although the 165 kGy dose caused a greater pH decrease by even more, by 0.15 units, the amount and character of binding points in such wool are already completely different in comparison with the lowest doses. 

The increase pH in water bath for 20 and 24 kGy wool compared with the 0 kGy sample can be interpreted as a consequence of OH^-^ groups coming from dissociation of water forced by present S-sulphoxides having a strong tendency to create H-bonds as mentioned above. In addition, the SO_4_^2−^ hydrolysis (Equation (4)) draws protons off enhancing the water dissociation, thus increasing the OH^-^ concentration, too (Equation (11)):

2 H_2_O ⇆ H_3_O^+^+ OH^−^ (11)

Consequently, the presence of primary S-oxidized species (as S-sulphonate R-S-SO_3_^–^, cystine monooxide R-S-SO^−^, cystine dioxide R-S-SO_2_^−^ ) generated by low doses [16,26] and being strong acceptors of H-bonds are responsible for remarkable change of pH value in the water bath after watering of 20 and 24 kGy dosed wool.

## 3. Materials and Methods

### 3.1. Materials 

Copper (II) sulphate pentahydrate CuSO_4_·5H_2_O p.a. was supplied by Centralchem (Bratislava, Slovakia). Test solutions for the sorption experiments were prepared by diluting the corresponding stock solution with demineralized water. 

The standard solution of Cu for AAS calibration (1000 mg.dm^−3^ in 3% HNO_3_) was supplied by Agilent Technologies (Santa Clara, CA, USA). Nitric acid 67% ANALPURE^TM^ for trace analysis to dilute the standard and wash the AAS spectrometer was obtained from Analytika (Prague, Czech Republic). 

The sheep wool came from spring sheep-shearing of a Tsigai-Suffolk crossbreed bred in Middle Slovakia. The samples were taken from various sites randomly and the fibre thickness was within the range of 27–33 μm. 

### 3.2. Sheep Wool Scouring and Irradiation 

After removing crude impurities manually, the wool was scoured repeatedly in warmish tap water until the rinse water was clean. An ultrasonic bath of 5 litre volume was used for the finishing scouring so that about 12 g of wool put in a netted pouch was washed in 40 °C tap water in the bath during a 10 min period. Then the water was exchanged and the washing was repeated again. Finally, the wool was rinsed with 5 litres of demineralized water. After the pouch was removed, and the trapped water ran off, the sample was dried in a laboratory oven at 40 °C for 24 h. Such dried wool was stored under routine laboratory conditions. The samples put in separate unsealed polyethylene pouches and placed into carton boxes were irradiated in a UELR-5-1S linear electron accelerator (FGUP NIIEFA, Petersburg, Russia) of Progresa Final SK operator. The process parameters were as follows: installed energy of 5 MeV, intensity of 200 μA, mean power of 1 kW and mean dose rate of 750 kGy/h. The doses applied were 0–20–24–48–100–165 kGy repeating 100 kGy cycles plus needed supplementing dose, if necessary. Between individual irradiation cycles, the samples were allowed to cool down for 30 min to maintain a temperature below 50 °C. The absorbed doses were checked dosimetrically. After being irradiated the samples were kept under usual laboratory conditions.

### 3.3. Batch Sorption Experiments 

The sorption experiments were conducted with Cu(II) solutions applying concentrations in the range of (12.5–80) mmol Cu·dm^−3^. After being cut to 3–5 mm, wool fibres (0.2 g) were placed into a small glass cup with a cap and the testing solution of 12 cm^3^ in volume was added. A thorough wetting of the wool was ensured immersing it into the solution using a glass rod. The content of the glass cup was shaken for first 6 h at room temperature on a laboratory horizontal shaker (TE, Kavalier, Sázava, Czech Republic) and then kept in static mode for next 18 h. Then the remaining solution was filtered through KA5 filter paper and used for determination of residual Cu(II). Every sorption experiment was carried out in triplicate. 

The sorption capacity was calculated using the following Equation:

(12) $$S=\left(x_{1}-x_{2}\right)/m$$

where S is the sorption capacity defined as the mass of sorbate in mg per 1 g of the sorbent for individual wool samples when particular testing solution is applied in specified concentration, ×1 is the mass of the Cu(II) added in the initial solution (mg), ×2 is the residual mass of the Cu(II) in the solution after its contact with the wool sample (mg), m is the mass of wool sample taken for analysis (g).

### 3.4. Determination of Residual Cu(II) Concentration 

Atomic absorption spectrometry (240 FS AAS, Agilent Technologies) was utilized for sorption experiments applying low concentrations of Cu(II) in the range 0.2–0.8 mmol·dm^−3^. The operative parameters were as follows: wavelength emitted by lamp of 324.8 nm, flow acetylene/air of 2.0/13.5 dm^−3^·min^−1^, concentration of Cu(II)-calibration solutions of 4–6–10 mg·dm^−3^, solution used to optimize absorbance Cu-signal to 0.2 with concentration of 1.5 mg·dm^−3^.

Higher Cu(II) concentrations of 10–100 mmol·dm^−3^ added into the bath was determined using visible spectrometry (Specord 50 Plus, Analytik Jena, Jena, Germany) with 1 cm cell recording spectrum within (450–1000) nm. The spectrum was taken from the filtered solution and Cu(II) content was estimated by means of the calibration curve. Concerning spectra, water extract from corresponding wool sample obtained under identical conditions such as Cu(II) residual solution was used as a blank for each series of the samples with identical absorbed dose. 

### 3.5. FTIR Spectral Measurements 

Fourier transform infrared spectroscopy-attenuated total reflectance (FTIR-ATR) measurements were performed with an NICOLET 8700^TM^ FTIR™ spectrometer (Thermo Scientific, Waltham, MA, USA) using a single bounce ATR accessory equipped with a Ge crystal. For transmission measurements the fibres were immersed directly into liquid nitrogen for 5–10 min and then ground in the ball mill. The ground powder in amount 0.9–1.9 mg was moulded into KBr pellets. The corresponding spectra were taken within whole middle infrared region (400–4000 cm^−1^) and normalized by converting to unit mass. In case of CuSO_4_ the quantity was 2.5 mg. For each measurement, the spectral resolution was 4 cm^−1^ and 64 scans were performed. The acquired spectra were analysed using the OMNIC™ v.8.1 spectroscopic software.

### 3.6. EPR Spectral Measurements

The EPR spectra of irradiated wool treated in solutions containing cupric ions were measured in solid state using an EMX EPR spectrometer (Bruker, Billerica, MA, USA) equipped with variable temperature unit. Cylindrical quartz tubes were used for measurements.

### 3.7. Measurement of pH

Value of pH in the bath was measured using an Orion2 Star pH-meter (Thermo Scientific) equipped with a Sen Tix 42 plastic electrode with temperature sensor. Applying double measuring the relative error did not exceed ± 0.5%. 

## 4. Conclusions

Sorption of Cu(II) onto sheep wool, both natural or modified by an accelerated electron beam, was investigated. Using higher Cu(II) concentrations of (10–100) mmol·dm^−3^ corresponding sorption isotherms with unexpected development were observed, showing extremes. The position of the extremes observed on the isotherms varies with the absorbed dose. FTIR transmission, ATR as well as EPR spectra indicate that Cu(II) creates complexes with both natural and irradiated wools. Formation of the complexes is considerable on surface of the fibre and near it. This potential varies with absorbed dose. Regarding the variety of suitable ligands (amines, imines, hydroxyls, amides) as well as cysteic and carboxyl acids present in modified or unmodified keratin, a large spectrum of products with coordinate bonds is generated. Considerable change of the secondary structure due to the electron beam affects the process, too. Since the ligands of a central Cu atom can come from different chains of keratin, complex links are formed linking them together (“crosslinking”) and formation of clusters occurs. The clusters form a barrier hindering access of further ions inside the fibre, which causes a decrease of total sorption of the cation. When available ligands on surface or near surface are spent, further Cu(II) cations diffusing inside need not bind only by a complexation (chelating) mechanism, but can form Cu(II)-salts with cysteic or carboxyl acids. Within this phase the isotherm can increase again. When diffusing Cu(II)-salts in the fibre bulk again obtain a proper position towards potential ligands inside, a new phase of complex generation occurs corresponding with a decrease of the isotherm in consequence of the formation of new clusters.
